# Supplementary material for: Anatomy of the first six months of COVID-19 vaccination campaign in Italy
Source: PLoS Comput Biol. 2022 May 25;18(5):e1010146. doi: 10.1371/journal.pcbi.1010146 (PMC9173644; doi:10.1371/journal.pcbi.1010146)
Supplement: S1 Text — In this supplementary material we present the model calibration procedure, additional analyses and extensions of our model. We also run extensive sensitivity analysis on the parameters used in the main text. (PDF) [file pcbi.1010146.s001.pdf]

Supplementary Material for *Anatomy of the first six months of COVID-19 Vaccination Campaign in Italy*

Nicolò Gozzi[1], Matteo Chinazzi[2], Jessica T. Davis[2], Kunpeng Mu[2], Ana Pastore y Piontti[2], Marco Ajelli[3,2], Nicola Perra[1,2,4], Alessandro Vespignani[2]

[1] Networks and Urban Systems Centre, University of Greenwich, UK
[2] Laboratory for the Modeling of Biological and Socio-technical Systems, Northeastern University, Boston, Massachusetts, USA
[3] Department of Epidemiology and Biostatistics, Indiana University School of Public Health, Bloomington, Indiana, USA
[4] School of Mathematical Sciences, Queen Mary University of London, London, United Kingdom

May 17, 2022

Contents

1 Model Definition **2**
1.1 Reproductive Number Calculation 4

2 Model Calibration **7**

3 Modeling non-pharmaceutical interventions: school closures **9**

4 Italian Regions **10**

5 Vaccine Hesitancy **12**

6 Different Transmissibility of Alpha Variant **13**

7 Increased Mortality for the Alpha Variant **15**

8 The Role of Asymptomatic and Recovered on Vaccinations **16**

9 Baseline with behavioral reactions **16**

10 Impact of vaccine protection delays distributions and second doses administration **18**

1 Model Definition

We report here the system of constitutive equations that defines the epidemic model. To simplify the notation we define the force of infection as $\lambda_k(t) = \beta \times s(t) \times \left\{ \sum_{k'} \frac{\hat{C}_{kk'}(t)}{N_{k'}} \left[I_{k'} + (1 - VE_I)(I_{k'}^{V1} + I_{k'}^{V2}) \right] \right\}$, where β is the transmission rate, $s(t)$ is the seasonality factor, $\hat{C}_{kk'}(t)$ is the k, k' element of the contacts matrix with the reductions due to NPIs implemented, and the term in brackets is the probability of contacting an infectious individual in age group k' given the contact rates and the number of individuals per group. In the computational implementation, the model's equations are integrated through chain binomial processes in order to ensure the discrete and stochastic nature of the transitions among compartments. Namely, at each time step t the number of individuals in age group k and compartment X transiting to compartment Y is sampled from $Pr^{Bin}(X_k(t), p_{X_k \to Y_k}(t))$, where $p_{X_k \to Y_k}(t)$ is the transition probability. For the sake of readability we report below the ODE's version of the constitutive

equations for each age group k.

$$
\begin{aligned}
\dot{S_k} &= -\lambda_k S_k - (1+\psi)\lambda_k S_k - \Omega_{S_k}(t)\\
\dot{S_k^{V1}} &= -(1-VE_{S1})\lambda_k S_k^{V1} - (1-VE_{S1}^{VOC})(1+\psi)\lambda_k S_k^{V1} - \Delta_{V2}^{-1} S_k^{V1} + \Omega_{S_k}(t)\\
\dot{S_k^{V2}} &= -(1-VE_{S2})\lambda_k S_k^{V2} - (1-VE_{S2}^{VOC})(1+\psi)\lambda_k S_k^{V2}\\
\dot{L_{WT,k}} &= \lambda_k S_k - \epsilon L_{WT,k} - \Omega_{L_{WT,k}}(t)\\
\dot{L_{WT,k}^{V1}} &= (1-VE_{S1})\lambda_k S_k^{V1} - \epsilon L_{WT,k}^{V1} - \Delta_{V2}^{-1} L_{WT,k}^{V1} + \Omega_{L_{WT,k}}(t)\\
\dot{L_{WT,k}^{V2}} &= (1-VE_{S2})\lambda_k S_k^{V2} + \Delta_{V2}^{-1} L_{WT,k}^{V1} - \epsilon L_{WT,k}^{V2}\\
\dot{L_{VOC,k}} &= (1+\psi)\lambda_k S_k - \epsilon L_{VOC,k} - \Omega_{L_{VOC,k}}(t)\\
\dot{L_{VOC,k}^{V1}} &= (1-VE_{S1}^{VOC})(1+\psi)\lambda_k S_k^{V1} - \epsilon L_{VOC,k}^{V1} - \Delta_{V2}^{-1} L_{VOC,k}^{V1} + \Omega_{L_{VOC,k}}(t)\\
\dot{L_{VOC,k}^{V2}} &= (1-VE_{S1}^{VOC})(1+\psi)\lambda_k S_k^{V2} + \Delta_{V2}^{-1} L_{VOC,k}^{V1} - \epsilon L_{VOC,k}^{V2}\\
\dot{I_{WT,k}} &= \epsilon L_{WT,k} - \mu I_{WT,k}\\
\dot{I_{WT,k}^{V1}} &= \epsilon L_{WT,k}^{V1} - \mu I_{WT,k}^{V1}\\
\dot{I_{WT,k}^{V2}} &= \epsilon L_{WT,k}^{V2} - \mu I_{WT,k}^{V2}\\
\dot{I_{VOC,k}} &= \epsilon L_{VOC,k} - \mu I_{VOC,k}\\
\dot{I_{VOC,k}^{V1}} &= \epsilon L_{VOC,k}^{V1} - \mu I_{VOC,k}^{V1}\\
\dot{I_{VOC,k}^{V2}} &= \epsilon L_{VOC,k}^{V2} - \mu I_{VOC,k}^{V2}\\
\dot{R_{WT,k}} &= \mu(1-IFR_k) I_{WT,k} - \Omega_{R_{WT,k}}(t)\\
\dot{R_{WT,k}^{V1}} &= \mu\left[1-(1-VE_{M1})IFR_k\right] I_{WT,k}^{V1} - \Delta_{V2}^{-1} R_{WT,k}^{V1} + \Omega_{R_{WT,k}}(t)\\
\dot{R_{WT,k}^{V2}} &= \mu\left[1-(1-VE_{M2})IFR_k\right] I_{WT,k}^{V2} + \Delta_{V2}^{-1} R_{WT,k}^{V1}\\
\dot{R_{VOC,k}} &= \mu(1-IFR_k) I_{VOC,k} - \Omega_{R_{VOC,k}}(t)\\
\dot{R_{VOC,k}^{V1}} &= \mu\left[1-(1-VE_{M1}^{VOC})IFR_k\right] I_{VOC,k}^{V1} + -\Delta_{V2}^{-1} R_{VOC,k}^{V1} + \Omega_{R_{VOC,k}}(t)\\
\dot{R_{VOC,k}^{V2}} &= \mu\left[1-(1-VE_{M1}^{VOC})IFR_k\right] I_{VOC,k}^{V2} + \Delta_{V2}^{-1} R_{VOC,k}^{V1}\\
\dot{D_{WT,k}} &= \mu IFR_k I_{WT,k} - \Delta^{-1} D_{WT,k}\\
\dot{D_{WT,k}^{V1}} &= \mu(1-VE_{M1}) IFR_k I_{WT,k}^{V1} - \Delta^{-1} D_{WT,k}^{V1}\\
\dot{D_{WT,k}^{V2}} &= \mu(1-VE_{M1}) IFR_k I_{WT,k}^{V2} - \Delta^{-1} D_{WT,k}^{V2}\\
\dot{D_{VOC,k}} &= \mu IFR_k I_{VOC,k} - \Delta^{-1} D_{VOC,k}\\
\dot{D_{VOC,k}^{V1}} &= \mu(1-VE_{M1}^{VOC}) IFR_k I_{VOC,k}^{V1} - \Delta^{-1} D_{VOC,k}^{V1}\\
\dot{D_{VOC,k}^{V2}} &= \mu(1-VE_{M1}^{VOC}) IFR_k I_{VOC,k}^{V2} - \Delta^{-1} D_{VOC,k}^{V2}\\
\dot{D_{WT,k}^{o}} &= \Delta^{-1} D_{WT,k}\\
\dot{D_{WT,k}^{V1,o}} &= \Delta^{-1} D_{WT,k}^{V1}\\
\dot{D_{WT,k}^{V2,o}} &= \Delta^{-1} D_{WT,k}^{V2}\\
\dot{D_{VOC,k}^{o}} &= \Delta^{-1} D_{VOC,k}\\
\dot{D_{VOC,k}^{V1,o}} &= \Delta^{-1} D_{VOC,k}^{V1}\\
\dot{D_{VOC,k}^{V2,o}} &= \Delta^{-1} D_{VOC,k}^{V2}
\end{aligned} \tag{1}
$$

Where the terms $\Omega_{S_k}(t)$, $\Omega_{L_k}(t)$, $\Omega_{R_k}(t)$ describes the transition of individuals due to vaccination (1st dose). The number of individuals that receive the first dose at each time step is taken from real data [1], while the distribution of doses among age groups depend on the strategy considered. For simplicity we did not included in the model the delay Δ_V between inoculation and actual vaccine protection. In Tab. 1, 2, 3 we report a full list of parameters, their values and references.

Symbol	Meaning	Value	Ref
β	Transmission rate	fitted	[2]
ϵ	Inverse of latency period	1 / 4 days	[3, 4]
μ	Inverse of infectious period	1 / 2.5 days	[3, 4]
C_k, k'	Element of the contacts matrix	/	[5]
IFR	Infection Fatality Rate	Tab. 2	[6]
Δ	Number of days between $I \to D$ and death reporting	fitted	[7]
ψ	Increased transmissibility of Alpha VOC	0.5	[8, 9]
$s(t)$	Seasonality modulation	time-varying	[10]
VE_{S1}	Vaccine efficacy against infection (1 dose)	Tab. 3	[11]
VE_{S2}	Vaccine efficacy against infection (2 dose)	Tab. 3	[11]
VE_{M1}	Vaccine efficacy against death if infected (1 dose)	Tab. 3	[11]
VE_{M2}	Vaccine efficacy against death if infected (2 dose)	Tab. 3	[11]
VE_{S1}^{VOC}	Vaccine efficacy against infection with Alpha VOC (1 dose)	Tab. 3	[11]
VE_{S2}^{VOC}	Vaccine efficacy against infection with Alpha VOC (2 dose)	Tab. 3	[11]
VE_{M1}^{VOC}	Vaccine efficacy against deaths if infected with Alpha VOC (1 dose)	Tab. 3	[11]
VE_{M2}^{VOC}	Vaccine efficacy against deaths if infected with Alpha VOC (2 dose)	Tab. 3	[11]
VE_I	Vaccine efficacy against further transmission	40%	[11]
$\Delta_V 2$	Number of days for second inoculation	Tab. 3	[12]
Δ_V	Number of days between inoculation and vaccine protection	14 days	[11]

Table 1: Interpretation of different parameters and their values.

Age group	Infection Fatality Ratio (Salje et al)
0-9	0.001%
10-19	0.001%
20-24	0.005%
25-29	0.005%
30-39	0.02%
40-49	0.05%
50-59	0.2%
60-69	0.7%
70-79	1.9%
80+	8.3%

Table 2: Age stratified infection fatality rate from Ref. [6]

	VE_1 (VE_{S1})		VE_2 (VE_{S2})		Δ_{V2}
	**Wild Type**	**Alpha**	**Wild Type**	**Alpha**	
Pfizer	90% (80%)	50% (40%)	95% (90%)	90% (80%)	21 days
Moderna	90% (80%)	50% (40%)	95% (90%)	90% (80%)	28 days
AstraZeneca	70% (60%)	50% (40%)	80% (70%)	75% (65%)	90 days
Janssen	70% (60%)	50% (40%)	/	/	/

Table 3: **Vaccine efficacy.** We report the values of VE and VE_S in parenthesis. Using the relationship $VE = 1 - (1 - VE_S)(1 - VE_M)$ one can easily derive VE_{M1} and VE_{M2}.

1.1 Reproductive Number Calculation

We compute the reproductive number R_{t^*} (where t^* indicate the start of our simulations) of the model proposed using the Next Generation Matrix method [13]. We derive the R_{t^*} without the compartments related to the vaccinated and to the Alpha VOC. Indeed, our simulations start well before both the arrival of vaccines and of the variant. Therefore, these compartments do not affect the initial stage of the epidemic. For simplicity, we also do not consider seasonality as this factor only implies a rescaling $R_{t^*} \to s(t^*)R_{t^*}$. We consider the $2K$ equations (where K is the number of age groups) that describe the evolution in time of the number of infected individuals L and I:

$$\begin{aligned} \dot{L_k} &= \lambda_k S_k - \epsilon L_k \\ \dot{I_k} &= \epsilon L_k - \mu I_k \end{aligned} \tag{2}$$

Where λ_k is the force of infection for age group k previously introduced. In matrix notation:

$$\begin{bmatrix} \frac{dL_1}{dt} \\ \vdots \\ \frac{dL_K}{dt} \\ \frac{dI_1}{dt} \\ \vdots \\ \frac{dI_K}{dt} \end{bmatrix} = \begin{bmatrix} \lambda_1 S_1 \\ \vdots \\ \lambda_K S_K \\ 0 \\ \vdots \\ 0 \end{bmatrix} - \begin{bmatrix} \epsilon L_1 \\ \vdots \\ \epsilon L_K \\ \mu I_1 - \epsilon L_1 \\ \vdots \\ \mu I_K - \epsilon L_K \end{bmatrix} \tag{3}$$

$$\begin{bmatrix} \frac{d\theta_1}{dt} \\ \vdots \\ \frac{d\theta_K}{dt} \\ \frac{d\theta_{K+1}}{dt} \\ \vdots \\ \frac{d\theta_{2K}}{dt} \end{bmatrix} = \begin{bmatrix} F_1 \\ \vdots \\ F_K \\ 0 \\ \vdots \\ 0 \end{bmatrix} - \begin{bmatrix} V_1 \\ \vdots \\ V_K \\ V_{K+1} \\ \vdots \\ V_{2K} \end{bmatrix} \tag{4}$$

Then, we define the disease free equilibrium (DFE) for age group k as $(S_k, L_k, I_k, R_k) \simeq (N_k - \tilde{R}_k, 0, 0, \tilde{R}_k)$, where $\tilde{R}_k$ is the residual immunity from previous waves. We define the two matrices F and V as follows: $F_{ij} = \frac{dF_i}{d\theta_j}\big|_{DFE}$ and $V_{ij} = \frac{dV_i}{d\theta_j}\big|_{DFE}$. These can be written as:

$$F = \begin{bmatrix} 0 & \cdots & 0 & \beta\frac{(N_1-\tilde{R}_1)C_{11}}{N_1} & \cdots & \beta\frac{(N_1-\tilde{R}_1)C_{1K}}{N_K} \\ \vdots & \ddots & \vdots & \vdots & \ddots & \vdots \\ 0 & \cdots & 0 & \beta\frac{(N_K-\tilde{R}_K)C_{K1}}{N_1} & \cdots & \beta\frac{(N_K-\tilde{R}_K)C_{KK}}{N_K} \\ 0 & \cdots & 0 & 0 & \cdots & 0 \\ \vdots & \ddots & \vdots & \vdots & \ddots & \vdots \\ 0 & \cdots & 0 & 0 & \cdots & 0 \end{bmatrix} \tag{5}$$

$$V = \begin{bmatrix} \epsilon & \cdots & 0 & 0 & \cdots & 0 \\ \vdots & \ddots & \vdots & \vdots & \ddots & \vdots \\ 0 & \cdots & \epsilon & 0 & \cdots & 0 \\ -\epsilon & \cdots & 0 & \mu & \cdots & 0 \\ \vdots & \ddots & \vdots & \vdots & \ddots & \vdots \\ 0 & \cdots & -\epsilon & 0 & \cdots & \mu \end{bmatrix} \tag{6}$$

The reproductive number is defined as $R_t = \rho(FV^{-1})$, where $\rho(\cdot)$ indicates the spectral radius (i.e., leading eigenvalue). First, we compute V^{-1}. We recognize that V and F can be written in blocks as:

$$F = \begin{bmatrix} 0 & \beta\tilde{C} \\ 0 & 0 \end{bmatrix}, V = \begin{bmatrix} \epsilon\mathbb{1} & 0 \\ -\epsilon\mathbb{1} & \mu\mathbb{1} \end{bmatrix} \tag{7}$$

Where $\tilde{C}$ is the contacts matrix weighted by the relative susceptible population in different age groups (i.e. $\tilde{C_{ij}} = \frac{N_i - \tilde{R}_i}{N_j} C_{ij}$), $\mathbb{1}$ is the $K \times K$ identity matrix, and 0 indicates a $K \times K$ matrix with zero elements. The inverse of V is:

$$V^{-1} = \begin{bmatrix} 1/\epsilon\mathbb{1} & 0 \\ 1/\mu\mathbb{1} & 1/\mu\mathbb{1} \end{bmatrix} \tag{8}$$

The next step consists in computing the product FV^{-1}:

$$FV^{-1} = \begin{bmatrix} 0 & \beta\tilde{C} \\ 0 & 0 \end{bmatrix} \begin{bmatrix} 1/\epsilon\mathbb{1} & 0 \\ 1/\mu\mathbb{1} & 1/\mu\mathbb{1} \end{bmatrix} = \begin{bmatrix} \beta\tilde{C}/\mu & \beta\tilde{C}/\mu \\ 0 & 0 \end{bmatrix} \tag{9}$$

Finally, we are left with finding the spectral radius of FV^{-1} (i.e., its largest eigenvalue). The eigenvalue problem can be written as $det(FV^{-1} - \lambda \mathbb{1}) = 0$. Given the structure of FV^{-1}, and since we are interested in non-trivial solutions ($\lambda \neq 0$), the problem reduces to:

$$det[\beta \tilde{C}/\mu - \lambda \mathbb{1}] = 0 \tag{10}$$

Therefore $R_{t^*} = \rho(FV^{-1}) = \beta\rho(\tilde{C})/\mu$. In the main text we assumed that $\tilde{R}_k << N_k$, therefore we approximate the disease free equilibrium as $(S_k, L_k, I_k, R_k) \simeq (N_k, 0, 0, 0)$. This assumption holds in our case since the initial number of recovered across all age brackets is generally smaller than 10% of the total population.

2 Model Calibration

We calibrate the free parameters of the model using an Approximate Bayesian Computation (ABC) technique [14, 15]. We define the prior distributions of the free parameters $P(\theta)$, a number accepted sets N, an error metric $m(E, E')$, and a tolerance δ. We start sampling a set of parameters θ from $P(\theta)$ and generate an instance of the model using these parameters. Then, using the chosen error metric we compare an output quantity E' of the model with the corresponding real quantity E: if $m(E, E') < \delta$ then we accept the set θ, otherwise we reject it. We repeat this accept/reject step until N parameter sets are accepted. The empirical distribution of the accepted sets is an approximation of their real posterior distribution. We produce model's projections sampling parameters sets from the pool of accepted sets and generating an ensemble of possible epidemic trajectories. We then compute median and confidence intervals on this ensemble. In this work, we consider the following free parameters and priors:

- β, we explore values of the transmission rate parameter such that the associated $R_t = \rho(\tilde{C})s(t)\frac{\beta}{\mu}$ on the first simulation day $\sim U(1.2, 2.0)$ [2] (where $\tilde{C}$ is the contacts matrix weighted by the relative population in different age groups and $\rho(\cdot)$ is the leading eigenvalue; see Sec. 1.1);
- $\Delta \sim U(14, 25)$. The calibration range is informed by Ref. [7];
- $\alpha_{min} \sim U(0.5, 1.0)$, in doing so we explore values from strong (0.5) to absent (1.0) seasonality;
- we select initial conditions from an ensemble of realistic estimates from GLEAM. Each estimate has the number of individuals of different age groups in different compartments (S, L, I, R) on the start of the simulation (2020/09/01).

The model is calibrated separately for each of the six regions considered. We set the calibration period to 2020/09/01-2021/07/05. We consider weekly deaths as output quantity and the weighted mean absolute percentage error (wMAPE) as error metric. We also set the number of accepted sets $N = 3000$ and the tolerance $\delta = 0.40$ for Sicily and Sardinia and $\delta = 0.35$ for the remaining regions. For the different basins, we represent in Fig. 1 the number of weekly real and simulated deaths (median and 90% CI). We obtain a median wMAPE of 0.23 for North West, 0.26 for North East, 0.25 for Center, 0.18 for South, 0.30 for Sicily, and of 0.40 for Sardinia. In the Supplementary Information we report the posterior distributions of the free parameters obtained through the ABC calibration.

In Fig. 2 are shown the posterior distributions of the parameters calibrated through the ABC rejection algorithm [14, 15] for each region. In particular, we report the effective reproductive number R_t on the first simulation day, the delay (expressed in days) in deaths Δ, the seasonality parameter α_{min}, and the initial number of infected (I plus L individuals) and recovered per $100'000$ at the start of the simulation.

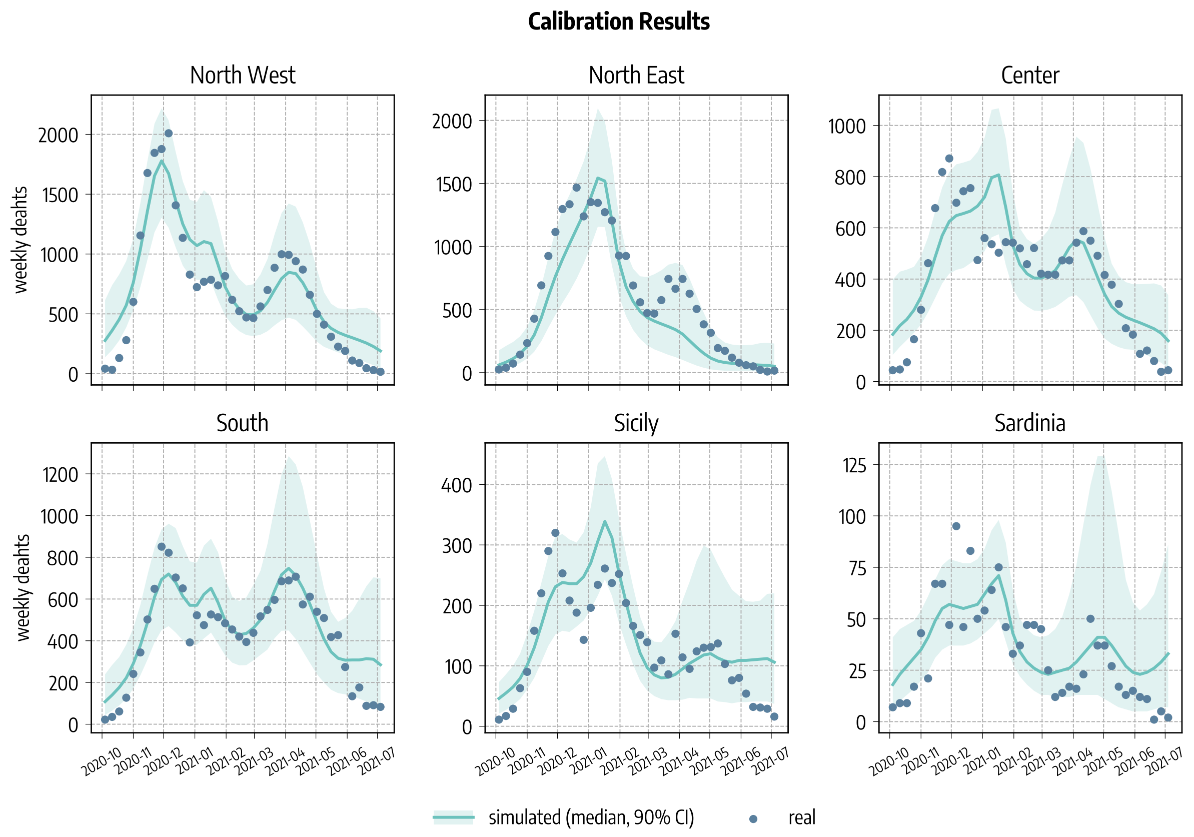

Figure 1: **Calibration Results**. Simulated (median and 90% CI) and observed cumulative number of deaths.

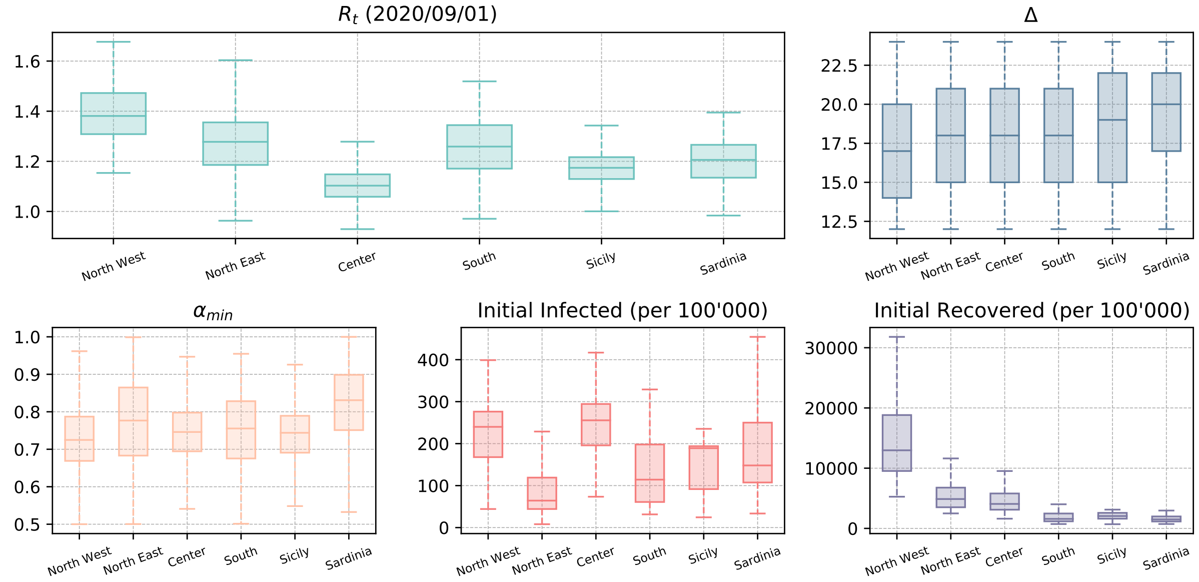

Figure 2: **Posterior distributions of parameters obtained through ABC calibration**. Each boxplot summarises the distribution of the parameter showing median, interquartile range (IQR), and *minimum* and *maximum* values defined respectively as $Q1 - 1.5IQR$ and $Q3 + 1.5IQR$ (where $Q1$ and $Q3$ are the first and third quartile).

3 Modeling non-pharmaceutical interventions: school closures

We model variations in contacts in schools as follows. First, we identify two phases. Before 2020/11/06, interventions from the Italian Government were mostly at the national level and different regions followed the same rules and recommendations. During this first phase, we use data from the Oxford COVID-19 Government Response Tracker [16] to model contacts variation in schools. We consider three indexes from this report about school closure ($school_{idx}$), workplace closure ($work_{idx}$), and stay at home requirements ($stayhome_{idx}$). These indexes vary daily and are provided at the national level. They can assume discrete values from 0 (no measures) to 3 (maximum restrictions). We define the parameters $w_s(t)$ and $\alpha(t)$ as follows:

1. $school_{idx}(t) = 0 \rightarrow w_s(t) = 1.0,\ \alpha(t) = 1.0$
2. $school_{idx}(t) = 1 \rightarrow w_s(t) = 0.5,\ \alpha(t) = 1.0$
3. $school_{idx}(t) = 2 \rightarrow w_s(t) = 1.0,\ \alpha(t) = 0.3444$
4. $school_{idx}(t) = 3$ and NOT$(work_{idx}(t) \geq 2$ and $stayhome_{idx}(t) \geq 2) \rightarrow w_s(t) = 1.0,\ \alpha(t) = 0.3444$
5. $school_{idx}(t) = 3$ and $(work_{idx}(t) \geq 2$ and $stayhome_{idx}(t) \geq 2) \rightarrow w_s(t) = 0.0,\ \alpha(t) = 1.0$

Finally, we model the impact of school closing on contacts on day t multiplying the school layer C^{school} (the elements describing contacts between $0-24$ years old individuals of the overall contacts matrix C) by $w_s(t)$ and by $\alpha(t)$. The two indexes are introduced to modify contacts that the younger age groups may have at school but also in other settings. The idea behind our approach is to define increasingly higher risk levels according to the policies implemented. The first case, for example, refers to a situation with no restrictions in place, therefore contacts in schools are not affected. When instead $school_{idx}(t) = 1$ some restrictions are in place, school closing or hybrid teaching mode and additional NPIs in classroom are recommended resulting in significant differences compared to normal operations [16]. We assume this would result in significant reduction of contacts in school. When $school_{idx}(t) = 2$ we introduce a reduction factor on the contacts of $0-24$ which is taken from Ref. [17], where authors studied the impact of school closure on contacts patterns among younger population. In the fourth case, strict closure policies on school are in place ($school_{idx}(t) = 3$) but younger age groups may still meet in different context apart from school (i.e., the work from home and the stay at home indexes are not at the maximum value). We assume that this would result in the same contacts reduction of the previous cases. Finally, when closure is required at all levels we send to zero the layer of contacts in schools.

After 2020/11/06, the Italian government introduced a tier system in which different NUTS2 regions were assigned to one of four risk levels (from 0, lowest risk, to 4 highest risk) [18]. Different tiers are associated with increasingly more restrictive measures. Because of our spatial resolution, first we define the risk tier at the NUTS1 level as the weighted average (according to the population) of the risk levels of the component NUTS2 regions (rounded to the nearest integer). For example, for NUTS1 region R, the risk tier at time t is:

$$TIER_R(t) = \left\lfloor \frac{\sum_{r \in R} p_r * TIER_r(t)}{\sum_{r \in R} p_r} \right\rceil \tag{11}$$

Where the summation runs over all NUTS2 regions r which are part of NUTS1 region R, and p_r is the population of NUTS2 region r taken from official sources [19]. Finally, we define the parameters $w_s(t)$ and $\alpha(t)$ similarly to what we did before for the first phase:

1. $TIER_R(t) = 0 \rightarrow w_s(t) = 0.5,\ \alpha(t) = 1.0$
2. $TIER_R(t) = 1 \rightarrow w_s(t) = 0.5,\ \alpha(t) = 1.0$
3. $TIER_R(t) = 2 \rightarrow w_s(t) = 1.0,\ \alpha(t) = 0.3444$
4. $TIER_R(t) = 3 \rightarrow w_s(t) = 0.0,\ \alpha(t) = 1.0$

Then the contacts matrix for school on day t is again obtained by the multiplying C^{school} by $w_s(t)$ and by $\alpha(t)$ the contact rates between $0-24$ years old individuals.

4 Italian Regions

In Fig. 3 we report some demographic and epidemiological characteristics of the different Italian regions under study. In Fig. 3A we show the distribution (%) of individuals in different age groups. We observe that, in general, the population is younger in South Italy and Sicily even tough the difference with other regions is extremely limited. Fig. 3B reports the estimated date (median and 90% CI) of the first Alpha VOC importation estimated by the Global Epidemic and Mobility model (GLEAM [10, 20, 21]). Importations are highly synchronized across regions. Indeed, across the board, the initial seeding of the variant happened throughout October 2020, and slightly later (early November 2020) in Sardinia. Fig. 3C shows the attack rate (i.e., cumulative incidence per 100) as reported by official surveillance and as estimated by GLEAM on the 2020/09/01 (start of our simulations). We notice good accordance between modeling estimates and official data. As expected, estimated attack rates are generally much higher than reported ones ($\sim 10\times$). We notice significant differences among regions. Sicily, Sardinia, and South Italy feature a smaller attack rate with respect to Center, and especially North East and North West. We also characterize regions according to the unfolding of the vaccine rollout in Fig. 3D, where it is reported the cumulative number of first doses administered per 100. The evolution of the number of doses given is extremely similar across regions with some difference at the end of the period of observation (e.g., Sicily has a slightly smaller number of doses given respect to other regions). Finally, Fig. 3E and Fig. 3F shows population size and density. Northwest is the most numerous and densely populated region. The number of individuals and their density in North East, South, and Center are comparable. Finally, Sicily and Sardinia are less populated (they are NUTS2 regions), but while Sardinia is also less densely populated, Sicily has a high population density (similar to Northeast, Center, and Sicily).

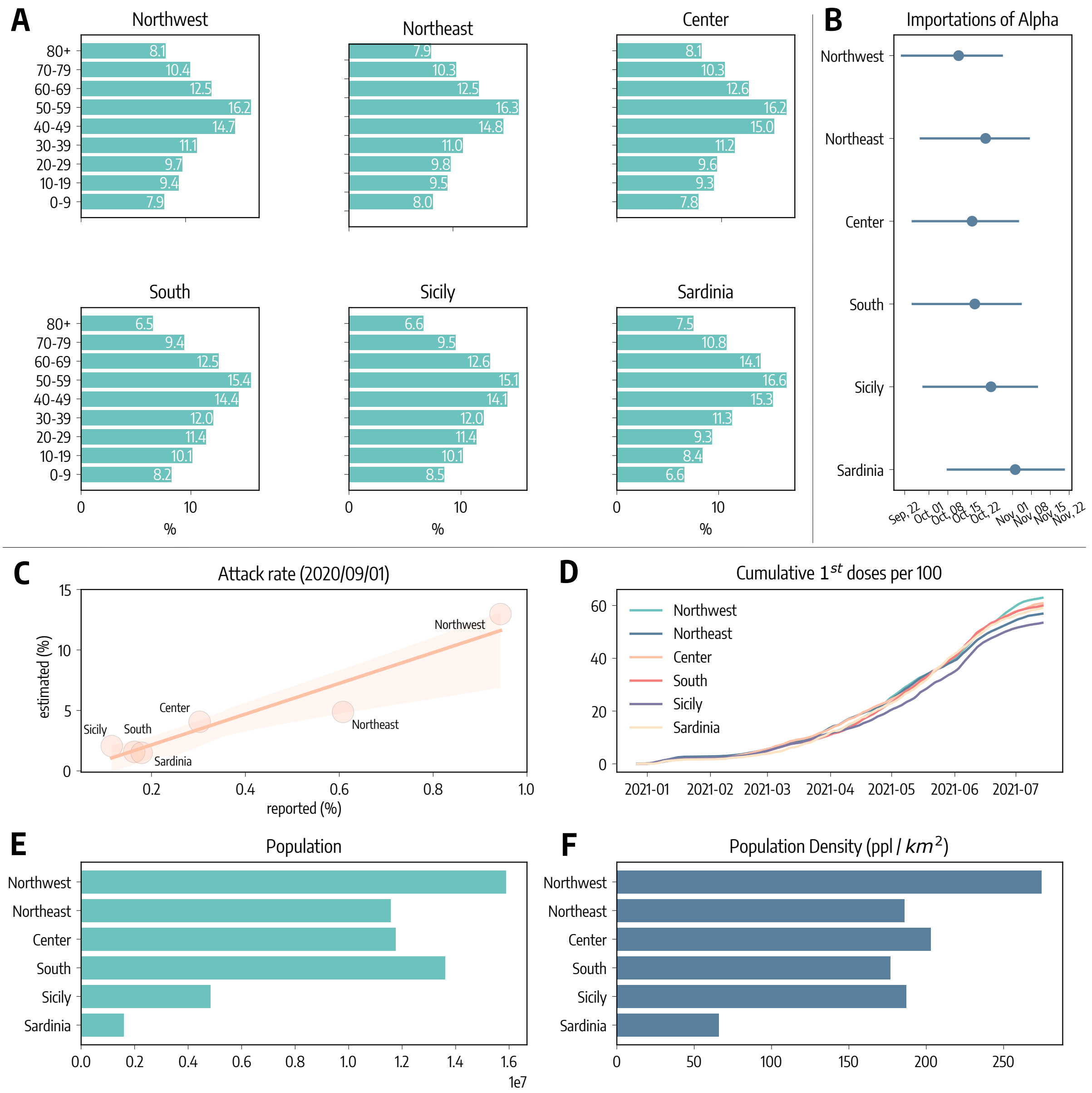

Figure 3: **Characteristics of Italian regions under study**. A) Distribution of individuals among age groups. B) Date of estimated first importation of Alpha VOC (median and 90% CI). C) Attack rate on 2020/09/01 (start of the simulation) as reported by official surveillance and as estimated by modeling. D) Cumulative number of first doses administered. E) Population size. F) Population density.

5 Vaccine Hesitancy

In the main text we assumed that in the counterfactual scenarios (*strategy 2* and *strategy 3*) all individuals are willing to receive the vaccines. Despite Italy showing very high rates of vaccine acceptance (as of 2021/10/19 85.7% received at least one dose [22]), this is an optimistic assumption. Therefore, we repeat here part of the analyses considering that a fraction of the population do not want to receive the vaccine. In Fig. 4 we show the the total number of averted deaths and infections (median and IQR) for *strategy 2* (prioritizing the elderly) and *3* (prioritizing the younger) for different values of hesitancy (i.e., the percentage of the population that refuses vaccines). We assume that hesitant individuals are homogeneously distributed among age groups. The horizontal dashed lines indicate the number of averted deaths/infections for the two strategies in the case of no hesitancy as presented in the main text. We observe, overall, a negligible effects of hesitancy. The explanation for such a small impact is that, as of 2021/07/05, *only* 58% of the population received at least one dose of vaccine in Italy. Therefore, the number of doses distributed in our simulations are not high enough to make the values of hesitancy considered significant. This also explains why we only observe an impact on model's outputs when considering averted deaths by *strategy 2*. In this scenario, we notice a decline in the number of averted deaths as the value of hesitancy grows. Indeed, since in this case we proceed in strictly decreasing age order between 50-80+, the values of hesitancy can play a role for older age groups that also feature higher IFRs.

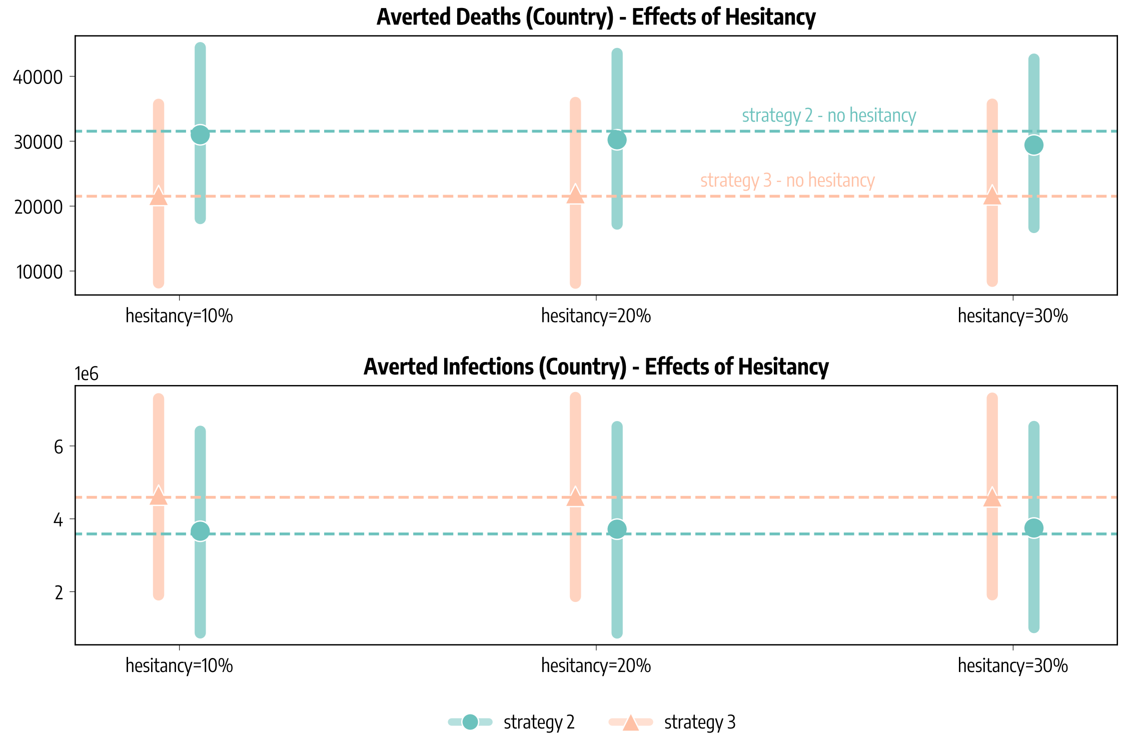

Figure 4: **Effects of vaccine hesitancy**

6 Different Transmissibility of Alpha Variant

In the main text we assumed the variant Alpha to be 50% (i.e., $\psi = 0.5$) more infectious than the wild type circulating previously, in line with available estimates [8, 9]. Nonetheless, we provide a sensitivity analysis to the results considering different values of ψ. In particular, we consider here $\psi = 0.3$ and $\psi = 0.6$. We repeat the calibration step for the two additional values of ψ. We note a need to increase the ABC tolerance in the case of $\psi = 0.6$ for convergence reasons. In Tab. 4 we report the obtained wMAPE between simulated and reported weekly deaths for the three values of ψ. We notice how the calibration with $\psi = 0.5$ gives in general better results.

	**wMAPE**		
	$\psi = 0.3$	$\psi = 0.5$	$\psi = 0.6$
North West	0.33	0.23	0.28
North East	0.32	0.26	0.23
Center	0.32	0.25	0.32
South	0.28	0.18	0.27
Sicily	0.29	0.30	0.46
Sardinia	0.33	0.40	0.57

Table 4: **wMAPE between simulated and reported weekly deaths for different ψ**

In Fig. 5A we show averted deaths and infections at the subnational and national level. We observe how figures are lower when considering $\psi = 0.3$. This is expected. Indeed a less transmissible variant causes a milder wave in our simulations with no vaccine and therefore the difference with the simulations with vaccine administered is smaller. For the opposite reason, the number of averted deaths and infections is higher, instead, in the case of $\psi = 0.6$. At the national level, we estimate vaccines would have averted $12,896$ (IQR: $[7,002 - 19,254]$) deaths for $\psi = 0.3$ and $39,242$ (IQR: $[24,539 - 53,543]$) with $\psi = 0.6$. As discussed in the main for $\psi = 0.5$ we have instead $29,350$ (IQR: $[16,454 - 42,826]$). Similarly, the estimated number of averted infections would have been $2,383,158$ (IQR: $[748,270 - 4,130,513]$) with $\psi = 0.3$ and $4,815,666$ (IQR: $[2,411,182 - 7,207,004]$) with $\psi = 0.6$. For $\psi = 0.5$ we have instead $4,256,332$(IQR: $[1,675,564 - 6,980,070]$).

In Fig. 5B we report the national prevalence of Alpha for the three values of ψ. When $\psi = 0.5$, we get a $wMAPE$ of 0.34 and a Pearson correlation coefficient $\rho = 0.84$ ($p < 0.001$) between the simulated prevalence and the one reported by genomic surveillance [23]. When $\psi = 0.3$ we get poorer results ($wMAPE = 0.53$, $\rho = 0.70$), and when $\psi = 0.6$ we get slightly better performance ($wMAPE = 0.31$, $\rho = 0.86$).

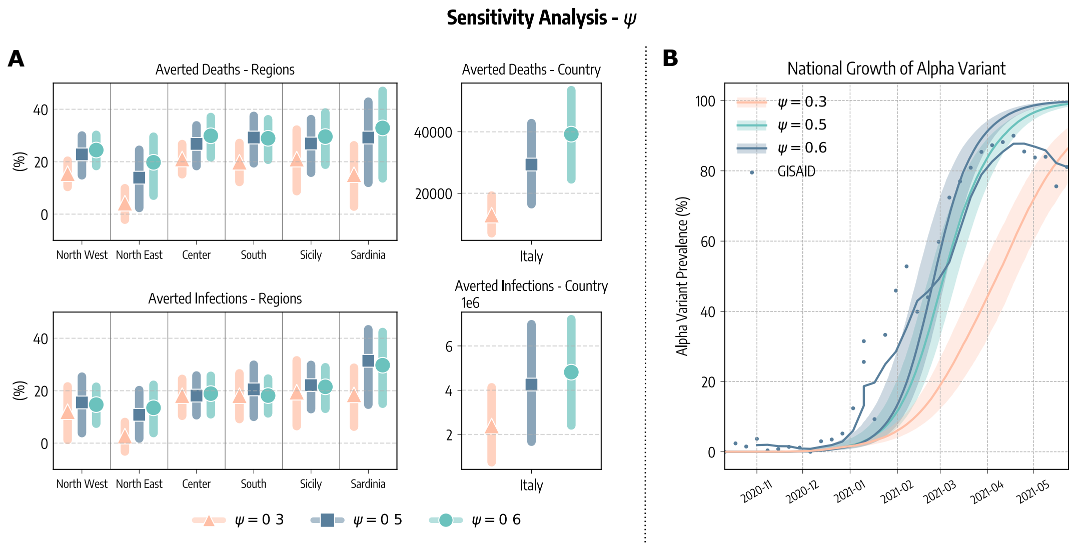

Figure 5: **Sensitivity analysis on ψ.** A) Averted deaths and infections at subnational and national level for different values of ψ. B) National prevalence of the Alpha variant as simulated with different ψ and as reported by genomic surveillance.

7 Increased Mortality for the Alpha Variant

In the main text we assumed the same Infection Fatality Ratio (IFR) for both the wild type and the Alpha variant. Nonetheless, previous works showed that the Alpha variant may also be associated with higher severity [24, 25]. To estimate how this impacts our findings we repeat here the analysis considering $IFR^{Alpha} = 1.5 IFR^{wildtype}$, where $IFR^{wildtype}$ is the one used in the main text and is taken from Ref. [6], and the 1.5 factor is in line with previous works [24, 25].

In Fig. 6 we show averted deaths and infections using the same IFR for wild type and Alpha and using the higher IFR for Alpha. We observe that our findings are not significantly impacted by the change of the IFR. Overall, when the higher IFR for Alpha is considered we estimate a slightly higher number of averted deaths. For example, at the national level we estimate vaccines averted $29,350$ (IQR: $[16,454 - 42,826]$) deaths when considering the same IFR for both strains, and $34,578$ (IQR: $[19,449 - 50,314]$) when considering a higher IFR for Alpha.

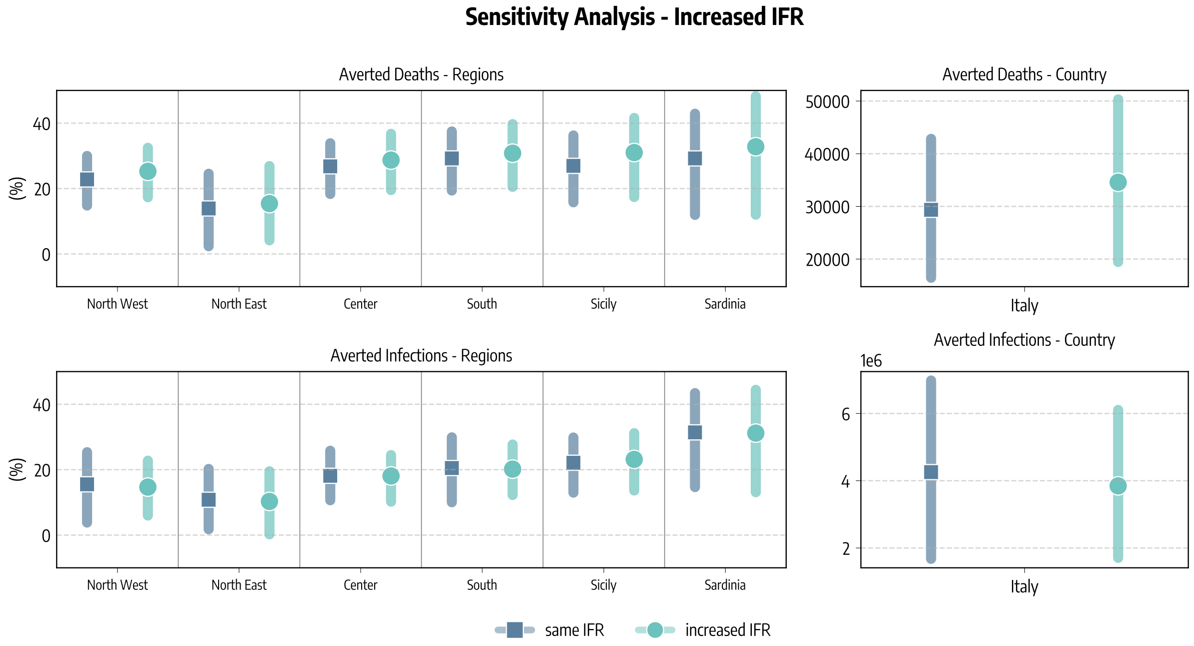

Figure 6: **Sensitivity analysis on Alpha variant IFR.** Averted deaths and infections at subnational and national level considering the same IFR for both wild type and Alpha and and a higher IFR for Alpha.

8 The Role of Asymptomatic and Recovered on Vaccinations

In the main text we assumed that all individuals but the infectious (I) can receive the vaccine. The real vaccination rollout in Italy, however, was more complex in terms of allocation. For example, individuals who recovered from the disease generally received only dose after at least three months from recovery and no more than six (then extended to twelve) [26]. At the same time, since vaccines were not officially preceded by a test, asymptomatic individuals may have received the vaccine as well. To assess how much the different allocation of vaccines among compartments can affect our result, we propose here the following analysis. In line with available estimates, we consider that 40% of all infections in our simulations are asymptomatic [27, 28]. Then, we assume that 40% of infectious individuals I (i.e., the asymptomatic) can also receive the vaccines. We also assume that only 40% of recovered can receive the vaccine. Said differently, we assume that symptomatic recovered do not receive the vaccine. We note how this is an extreme scenario in which all asymptomatic infectious are eligible for vaccine and none of the symptomatic recovered are. The goal here is indeed to test the extent to which our results are impacted by different choices of allocation of vaccines among compartments.

In Fig. 7 we show averted deaths and infections using the allocation of vaccines among compartments presented in the main text and the one just described. We see that the change of allocation does not significantly impact our findings. For example, the number of averted deaths with the new allocation is $35,404$ (IQR: $[21,577-50,200]$), while in the main text we obtained $29,350$ (IQR: $[16,454-42,826]$). The higher number of averted deaths maybe be due to the fact that, with the new allocation among compartments, vaccines are given only to a fraction of the recovered and as result more doses are administered to susceptibles individuals.

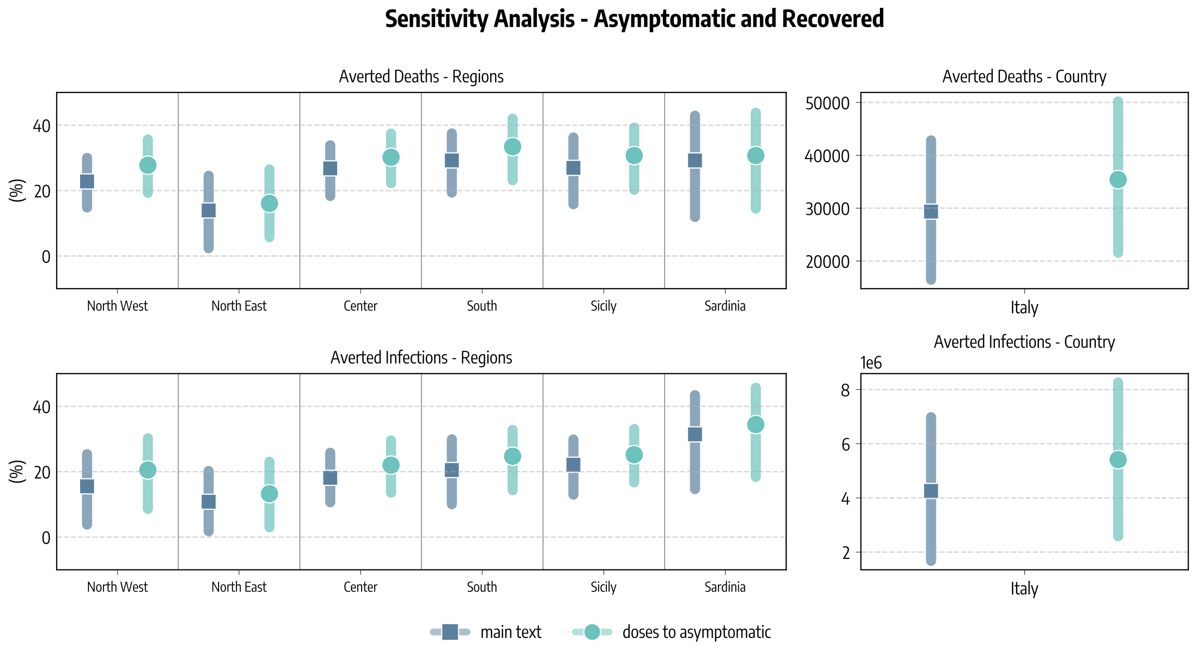

Figure 7: **Sensitivity analysis on allocation of vaccines among compartments.** Averted deaths and infections at subnational and national level considering the allocation among compartments proposed in the main text and considering also the role of asymptomatic and recovered.

9 Baseline with behavioral reactions

In the main text, the impact of vaccines is measured respect to a baseline that has everything equal but for the presence of vaccines. This approach allows us to isolate the impact of vaccines alone on the epidemic trajectory. Nonetheless, we acknowledge that the NPIs observed during the rollout were contingent to the improved epidemiological condition to which vaccines contributed in a substantial way. In the absence of vaccines, a worsening of the epidemiological conditions would have likely led to both

bottom-up (i.e., self-initiated) and top-down (i.e., government mandated) behavioral reactions [29]. As result, the baseline is a pessimistic estimate since it does not account for possible NPIs enforcement in response to rises in infections and fatalities in case of no vaccinations.

Here, we present a second baseline without vaccines in which we also model behavioral reactions. It is important to stress that we will never know what it could have happened in the spring of 2021 without vaccines, so any scenario aiming to account for behavioral changes it is speculative. Bearing this in mind we proceed as follows. First, we define a threshold for the introduction of tougher restrictive measures. As mentioned in the main text, on 2020/11/06 the Italian government introduced a risk-level system to adopt more targeted and timely countermeasures according to the local epidemic conditions. Therefore, for each of our six regions, we compute the average number of deaths on the days when the region was moved to the highest risk level (indicated as ''*red zone*"). We call this quantity $D_i^{red\ zone}$. Second, we compute the average contacts matrix $C_i^{red\ zone}$ observed during the days when in region i were in place the rules of the highest risk level. Finally, we follow this simple approach: in the simulation without vaccines administered, if the number of daily deaths in region i exceeds $D_i^{red\ zone}$ - and the region is not already in the highest risk level - we use the contacts matrix $C_i^{red\ zone}$ instead of the one computed following actual mobility change and policy interventions. We apply this check after 2021/03/01 since it is around this date that the epidemic trajectories with and without vaccines start to visibly diverge.

We stress how this threshold approach to model NPIs enforcement has many limitations. For example, the system of risk-levels developed in Italy depended on many parameters and not just on deaths [18]. Also, we consider a single threshold, while in reality multiple thresholds associated with increasingly higher risk levels were defined. As result, our approach is admittedly simplistic to represent the complexity of individual and collective response to epidemics [30]. Nonetheless, it captures the possible evolution of NPIs in a hypothetical scenario using historical real data and allows us to assess the impact of this on our findings.

In Fig. 8 we compare the evolution of weekly deaths as reported by official sources and as simulated by our model. We consider the case in which i) vaccines are administered according to the real data, ii) no vaccines are administered, iii) no vaccines are administered but NPIs are put in place to counter the uptick of cases and deaths. As expected, in this last scenario we observe less deaths with respect to the simple baseline considered in the main. Nonetheless, it is important to stress how also in this case the death toll is worse with respect to the case with vaccines.

In Tab. 5 and Tab. 6 we compare the number of averted deaths and infections by vaccines with respect to the two no-vaccine baselines in different regions. Not surprisingly, we observe that total number of averted deaths with respect to the new baseline is diminished, from 29,4064 (IQR: [16,571; 42,830]) to 12,676 (IQR: [2,795; 22,887]). At the same time, we also obtain a much lower number of averted infections (547,554 IQR: [−1,540,752; 2,701,933] respect to 4,306,913 IQR: [1,656,934; 6,990,557]).

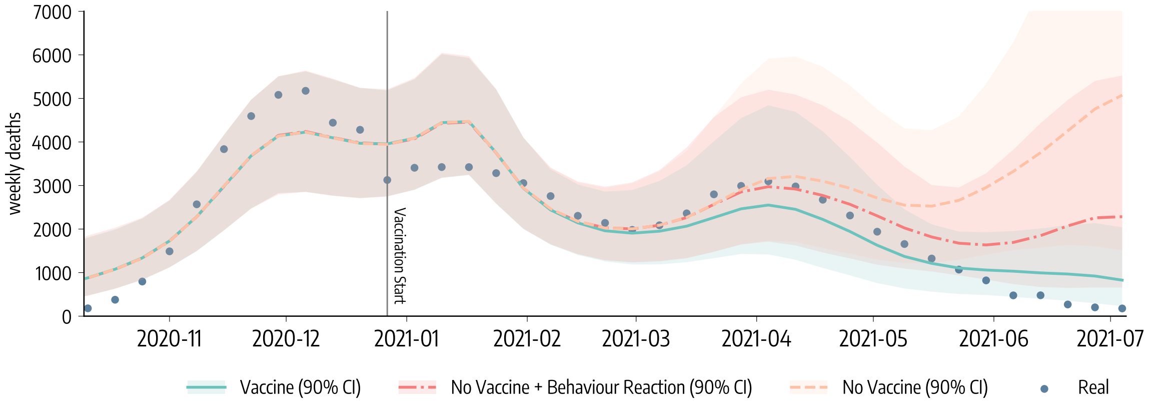

Figure 8: **Evolution of weekly deaths in Italy under different scenarios**. We show the number of weekly deaths in Italy as reported by official sources and s simulated by our model. We consider different vaccination scenarios: i) data-driven vaccination (solid line), ii) no vaccine (dashed line), and iii) no vaccine with possible behaviour reaction (dash-dotted line).

Averted Deaths (Median and IQR)		
	**No Vaccine Baseline**	**No Vaccine + Behavior Reaction Baseline**
North West	9047 [5525, 12492]	4672 [2052, 7480]
North East	3143 [750, 5961]	3128 [532, 5946]
Center	6431 [4077, 8736]	2469 [733, 4108]
South	7892 [4745, 11235]	1464 [-553, 3521]
Sicily	2263 [1246, 3310]	720 [56, 1387]
Sardinia	629 [228, 1095]	222 [-24, 444]
Italy (total)	29406 [16571, 42830]	12676 [2795, 22887]

Table 5: **Averted deaths - No vaccine baseline comparison**

Averted Infections (Median and IQR)		
	**No Vaccine Baseline**	**No Vaccine + Behavior Reaction Baseline**
North West	1011733 [269085, 1733792]	260,281 [-390544, 886551]
North East	506441 [43955, 993421]	467160 [11869, 1008596]
Center	875658 [460490, 1284045]	75351 [-228365, 386458]
South	1289381 [571882, 2044919]	-269856 [-745282, 201937]
Sicily	451476 [243112, 652044]	6086 [-134485, 155195]
Sardinia	172223 [68409, 282335]	8532 [-53945, 63195]
Italy (total)	4306913 [1656934, 6990557]	547554 [-1540752, 2701933]

Table 6: **Averted infections - No vaccine baseline comparison**

10 Impact of vaccine protection delays distributions and second doses administration

We propose an alternative version of the model in which we use a different distribution of the delays between inoculation and actual vaccine protection. As explained previously, in the main text we considered an average period of $\Delta_V = 14days$ between inoculation (of both 1st and 2nd dose) and vaccine protection. This means that a person receiving a dose on day t will be protected, on average, on day $t + \Delta_V$. In practice, individuals receiving the vaccine are moved to a temporary compartment, and transition to the final compartment of vaccinated with a rate $1/\Delta_V$. Consider the following example. The number of susceptible individuals receiving the first dose on day t is taken from real data. These individuals are moved to the temporary compartment S_r^{V1}. In this compartment they still have no vaccine protection, therefore they can get infected at the same rate as the susceptibles. Then, S_r^{V1} eventually transition to the vaccinated compartment S^{V1} with a rate $1/\Delta_V$. Also this transition is regulated by a binomial sample: $Pr^{Bin}(S_r^{V1}(t), 1/\Delta_V)$. The result is that, the times spent in the temporary compartment S_r^{V1} will be exponentially distributed with scale parameter $1/\Delta_V$ (mean Δ_V and variance Δ_V^2).

To assess the impact of this modeling choice, we propose here a model in which vaccine protection delays follow an Erlang distribution with shape parameter n [31]. Indeed, while the standard formulation of compartmental models assume that the time spent in different compartments is exponentially distributed, distributions might be narrower and centred around the mean [31]. The Erlang distribution is equivalent to a sequence of independent and identically distributed exponential distributions. Therefore, we modify the modeling setup including additional *temporary* compartments. Consider the previous example. In this new formulation of the model, susceptible individuals that receive the first dose move to the temporary compartment S_{r1}^{V1}, where they are still not protected by vaccines. Then, they transition to the next temporary compartment S_{r2}^{V1} at rate $n/(\Delta_V)$, where n is shape parameter and the number of temporary compartments. Finally, the individuals in S_{rn}^{V1} transition to the compartment of the vaccinated with one dose S^{V1}. Following this approach, we obtain that the time spent in the temporary compartments is distributed according to an Erlang distribution with shape parameter n and scale parameter $1/\Delta_V$ (mean Δ_V and variance Δ_V^2/n) [31]. In Fig. 9 we show the Erlang distribution for different values of n. We see that for $n = 1$ we get the exponential distribution, while for higher values the distribution is narrower and more peaked around the mean.

For simplicity, here we set $n = 2$. This implies defining two temporary compartments (S_{r1}^{V1} and S_{r2}^{V1} according to the previous example), in which each individual spends on average 7 days. As an additional sensitivity check to the modeling of vaccinations, here we also consider real data for second

doses. Indeed, in the main text we informed the number of daily first doses with real data from official sources, while we modeled the second inoculation introducing transitions happening at rate $1/\Delta_{V2}$, with Δ_{V2} depending on the vaccine manufacturer. This was done to simplify the model. Here, to assess the impact of this choice we inform the model also with the number of second doses administered per day by age group and manufacturer.

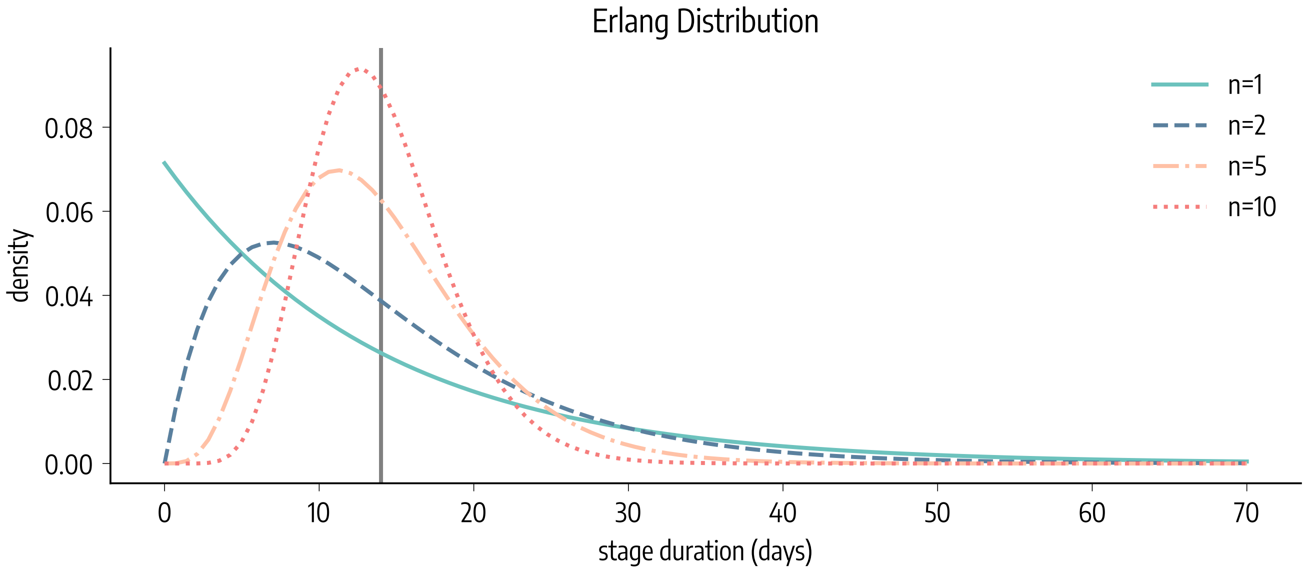

Figure 9: **Erlang distribution for several shape parameters**. Plot is adapted from Ref. [31]. Solid vertical line indicates the average stage duration $\Delta_V = 14 days$.

In Fig. 10 we compare different strategies in terms of averted deaths and infections using the modeling setup just described. We see that the main findings are unaltered. Indeed, the strategy prioritizing the elderly (*strategy 2*) generally implies more averted deaths, followed by the actual strategy and the strategy prioritizing 20-49 age groups (*strategy 3*). When considering averted infections, instead, the order is inverted: *strategy 3* guarantees less infections, followed by the actual strategy and *strategy 2*. The overall national figures are also very similar to those obtained in the main text. For example, we estimate here that the actual strategy averted $31,314$ (IQR: $[18,247 - 44,621]$) deaths compared to $29,350$ (IQR: $[16,454 - 42,826]$) as reported in the main text.

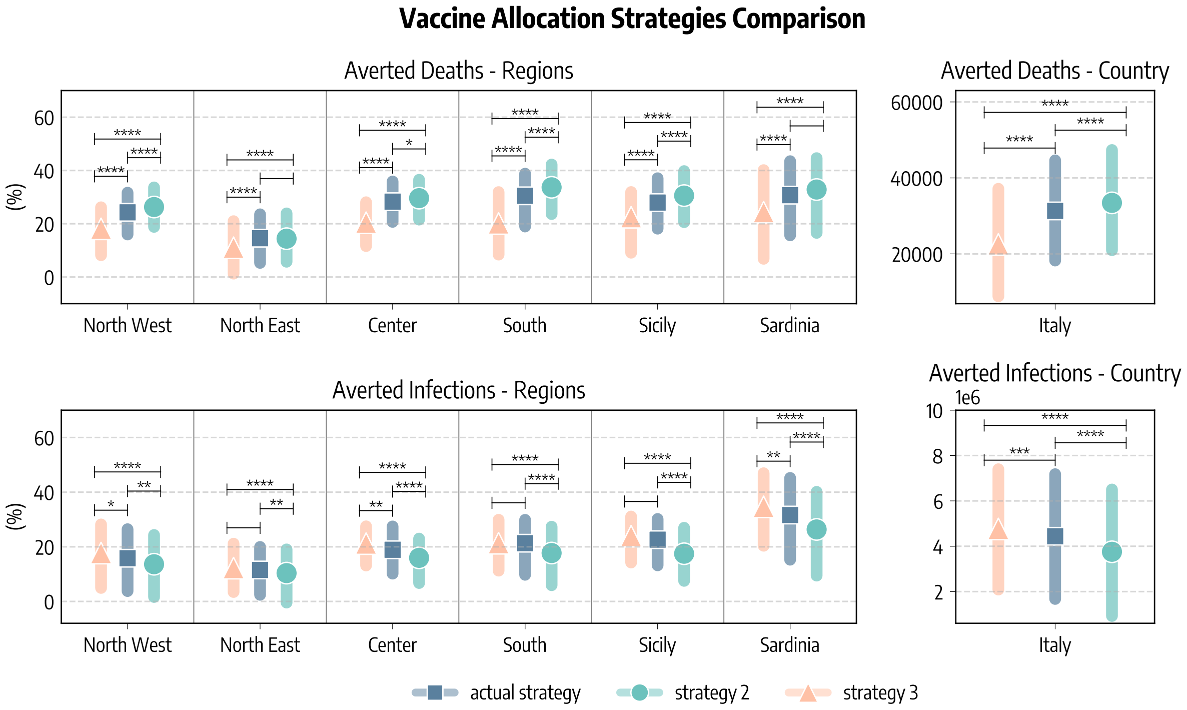

Figure 10: **Comparison of vaccine allocation strategies with Erlang distributed vaccine protection delays**. A) Averted COVID-19 deaths and infections, both at subnational and national level (medians and interquartile ranges are reported). B) Percentage reduction of the Infection Fatality Rate as of 2021/07/05. In all panels, *actual strategy* denotes an allocation strategy that follows the observed allocation as it unfolded during the pandemic, *strategy 2* considers the case where vaccines are allocated in decreasing age order starting from the 80+, *strategy 3* considers the case in which vaccines are first allocated to the age groups $20-49$ and then homogeneously to the rest of the population, and *no-vaccine* denotes the counterfactual scenario in which vaccines were not administered. In both panel we report the statistical significance of the Kruskal-Wallis H-test comparing different scenarios as follows: ****: $p_{value} \leq 10^{-4}$, ***: $10^{-4} < p_{value} \leq 10^{-3}$, **: $10^{-3} < p_{value} \leq 10^{-2}$, *: $10^{-2} < p_{value} \leq 0.05$, and otherwise blank if $p > 0.05$.

References

[1] Covid-19 Opendata Vaccini. https://github.com/italia/covid19-opendata-vaccini, 2021. Accessed: 2021-07-13.

[2] Temporal variation in transmission during the COVID-19 outbreak. https://epiforecasts.io/covid/, 2021. Accessed: 2021-07-12.

[3] Jantien A Backer, Don Klinkenberg, and Jacco Wallinga. Incubation period of 2019 novel coronavirus (2019-ncov) infections among travellers from wuhan, china, 20–28 january 2020. *Eurosurveillance*, 25(5), 2020.

[4] Stephen M. Kissler, Christine Tedijanto, Edward Goldstein, Yonatan H. Grad, and Marc Lipsitch. Projecting the transmission dynamics of sars-cov-2 through the postpandemic period. *Science*, 368(6493):860–868, 2020.

[5] Dina Mistry, Maria Litvinova, Ana Pastore y Piontti, Matteo Chinazzi, Laura Fumanelli, Marcelo F C Gomes, Syed A Haque, Quan-Hui Liu, Kunpeng Mu, Xinyue Xiong, M Elizabeth Halloran, Ira M Longini, Stefano Merler, Marco Ajelli, and Alessandro Vespignani. Inferring high-resolution human mixing patterns for disease modeling. *Nature Communications*, 12(1):323, 2021.

[6] Henrik Salje, Cécile Tran Kiem, Noémie Lefrancq, Noémie Courtejoie, Paolo Bosetti, Juliette Paireau, Alessio Andronico, Nathanaël Hozé, Jehanne Richet, Claire-Lise Dubost, Yann Le Strat, Justin Lessler, Daniel Levy-Bruhl, Arnaud Fontanet, Lulla Opatowski, Pierre-Yves Boelle, and Simon Cauchemez. Estimating the burden of SARS-CoV-2 in France. *Science*, 369(6500):208–211, 2020.

[7] Centers for Disease Control and Prevention, COVID-19 Pandemic Planning Scenarios. https://www.cdc.gov/coronavirus/2019-ncov/hcp/planning-scenarios.html#table-1, 2021. Accessed 2021/02/02.

[8] Nicholas G. Davies, Sam Abbott, Rosanna C. Barnard, Christopher I. Jarvis, Adam J. Kucharski, James D. Munday, Carl A. B. Pearson, Timothy W. Russell, Damien C. Tully, Alex D. Washburne, Tom Wenseleers, Amy Gimma, William Waites, Kerry L. M. Wong, Kevin van Zandvoort, Justin D. Silverman, CMMID COVID-19 Working Group, COVID-19 Genomics UK (COG-UK) Consortium, Karla Diaz-Ordaz, Ruth Keogh, Rosalind M. Eggo, Sebastian Funk, Mark Jit, Katherine E. Atkins, and W. John Edmunds. Estimated transmissibility and impact of sars-cov-2 lineage b.1.1.7 in england. *Science*, 372(6538), 2021.

[9] Erik Volz, Swapnil Mishra, Meera Chand, Jeffrey C Barrett, Robert Johnson, Lily Geidelberg, Wes R Hinsley, Daniel J Laydon, Gavin Dabrera, Áine O'Toole, Robert Amato, Manon Ragonnet-Cronin, Ian Harrison, Ben Jackson, Cristina V Ariani, Olivia Boyd, Nicholas J Loman, John T McCrone, Sónia Gonçalves, David Jorgensen, Richard Myers, Verity Hill, David K Jackson, Katy Gaythorpe, Natalie Groves, John Sillitoe, Dominic P Kwiatkowski, Cherian Koshy, Amy Ash, Emma Wise, Nathan Moore, Matilde Mori, Nick Cortes, Jessica Lynch, Stephen Kidd, Derek J Fairley, Tanya Curran, James P McKenna, Helen Adams, Christophe Fraser, Tanya Golubchik, David Bonsall, Mohammed O Hassan-Ibrahim, Cassandra S Malone, Benjamin J Cogger, Michelle Wantoch, Nicola Reynolds, Ben Warne, Joshua Maksimovic, Karla Spellman, Kathryn McCluggage, Michaela John, Robert Beer, Safiah Afifi, Sian Morgan, Angela Marchbank, Anna Price, Christine Kitchen, Huw Gulliver, Ian Merrick, Joel Southgate, Martyn Guest, Robert Munn, Trudy Workman, Thomas R Connor, William Fuller, Catherine Bresner, Luke B Snell, Amita Patel, Themoula Charalampous, Gaia Nebbia, Rahul Batra, Jonathan Edgeworth, Samuel C Robson, Angela H Beckett, David M Aanensen, Anthony P Underwood, Corin A Yeats, Khalil Abudahab, Ben E W Taylor, Mirko Menegazzo, Gemma Clark, Wendy Smith, Manjinder Khakh, Vicki M Fleming, Michelle M Lister, Hannah C Howson-Wells, Louise Berry, Tim Boswell, Amelia Joseph, Iona Willingham, Carl Jones, Christopher Holmes, Paul Bird, Thomas Helmer, Karlie Fallon, Julian Tang, Veena Raviprakash, Sharon Campbell, Nicola Sheriff, Victoria Blakey, Lesley-Anne Williams, Matthew W Loose, Nadine Holmes, Christopher Moore, Matthew Carlile, Victoria Wright, Fei Sang, Johnny Debebe, Francesc Coll, Adrian W Signell, Gilberto Betancor, Harry D Wilson, Sahar Eldirdiri, Anita Kenyon, Thomas Davis, Oliver G Pybus, Louis du Plessis, Alex E Zarebski, Jayna Raghwani, Moritz U G Kraemer, Sarah Francois, Stephen W Attwood, Tetyana I Vasylyeva, Marina Escalera Zamudio, Bernardo

Gutierrez, M Estee Torok, William L Hamilton, Ian G Goodfellow, Grant Hall, Aminu S Jahun, Yasmin Chaudhry, Myra Hosmillo, Malte L Pinckert, Iliana Georgana, Samuel Moses, Hannah Lowe, Luke Bedford, Jonathan Moore, Susanne Stonehouse, Chloe L Fisher, Ali R Awan, John BoYes, Judith Breuer, Kathryn Ann Harris, Julianne Rose Brown, Divya Shah, Laura Atkinson, Jack C D Lee, Nathaniel Storey, Flavia Flaviani, Adela Alcolea-Medina, Rebecca Williams, Gabrielle Vernet, Michael R Chapman, Lisa J Levett, Judith Heaney, Wendy Chatterton, Monika Pusok, Li Xu-McCrae, Darren L Smith, Matthew Bashton, Gregory R Young, Alison Holmes, Paul Anthony Randell, Alison Cox, Pinglawathee Madona, Frances Bolt, James Price, Siddharth Mookerjee, Manon Ragonnet-Cronin, Fabricia F Nascimento, David Jorgensen, Igor Siveroni, Rob Johnson, Olivia Boyd, Lily Geidelberg, Erik M Volz, Aileen Rowan, Graham P Taylor, Katherine L Smollett, Nicholas J Loman, Joshua Quick, Claire McMurray, Joanne Stockton, Sam Nicholls, Will Rowe, Radoslaw Poplawski, Alan McNally, Rocio T Martinez Nunez, Jenifer Mason, Trevor I Robinson, Elaine O'Toole, Joanne Watts, Cassie Breen, Angela Cowell, Graciela Sluga, Nicholas W Machin, Shazaad S Y Ahmad, Ryan P George, Fenella Halstead, Venkat Sivaprakasam, Wendy Hogsden, Chris J Illingworth, Chris Jackson, Emma C Thomson, James G Shepherd, Patawee Asamaphan, Marc O Niebel, Kathy K Li, Rajiv N Shah, Natasha G Jesudason, Lily Tong, Alice Broos, Daniel Mair, Jenna Nichols, Stephen N Carmichael, Kyriaki Nomikou, Elihu Aranday-Cortes, Natasha Johnson, Igor Starinskij, Ana da Silva Filipe, David L Robertson, Richard J Orton, Joseph Hughes, Sreenu Vattipally, Joshua B Singer, Seema Nickbakhsh, Antony D Hale, Louissa R Macfarlane-Smith, Katherine L Harper, Holli Carden, Yusri Taha, Brendan A I Payne, Shirelle Burton-Fanning, Sheila Waugh, Jennifer Collins, Gary Eltringham, Steven Rushton, Sarah O'Brien, Amanda Bradley, Alasdair Maclean, Guy Mollett, Rachel Blacow, Kate E Templeton, Martin P McHugh, Rebecca Dewar, Elizabeth Wastenge, Samir Dervisevic, Rachael Stanley, Emma J Meader, Lindsay Coupland, Louise Smith, Clive Graham, Edward Barton, Debra Padgett, Garren Scott, Emma Swindells, Jane Greenaway, Andrew Nelson, Clare M McCann, Wen C Yew, Monique Andersson, Timothy Peto, Anita Justice, David Eyre, Derrick Crook, Tim J Sloan, Nichola Duckworth, Sarah Walsh, Anoop J Chauhan, Sharon Glaysher, Kelly Bicknell, Sarah Wyllie, Scott Elliott, Allyson Lloyd, Robert Impey, Nick Levene, Lynn Monaghan, Declan T Bradley, Tim Wyatt, Elias Allara, Clare Pearson, Husam Osman, Andrew Bosworth, Esther Robinson, Peter Muir, Ian B Vipond, Richard Hopes, Hannah M Pymont, Stephanie Hutchings, Martin D Curran, Surendra Parmar, Angie Lackenby, Tamyo Mbisa, Steven Platt, Shahjahan Miah, David Bibby, Carmen Manso, and The COVID-19 Genomics U K (COG-UK) Consortium. Assessing transmissibility of SARS-CoV-2 lineage B.1.1.7 in England. *Nature*, 593(7858):266–269, 2021.

[10] Duygu Balcan, Bruno Gonçalves, Hao Hu, José J. Ramasco, Vittoria Colizza, and Alessandro Vespignani. Modeling the spatial spread of infectious diseases: The global epidemic and mobility computational model. *Journal of Computational Science*, 1(3):132 – 145, 2010.

[11] Julia Shapiro, Natalie E. Dean, Zachary J. Madewell, Yang Yang, M.Elizabeth Halloran, and Ira Longini. Efficacy estimates for various covid-19 vaccines: What we know from the literature and reports. *medRxiv*, 2021.

[12] Prevalenza e distribuzione delle varianti del virus SARS-CoV-2 di interesse per la sanità pubblica in Italia. `https://www.iss.it/documents/20126/0/reportBollettino+varianti+fino+al+19+maggio+2021.pdf/1e7218cc-c084-a7af-0a4c-6573acb3eba9?t=1621944222307`, 2021. Accessed: 2021-07-29.

[13] Pauline Driessche. Reproduction numbers of infectious disease models. *Infectious Disease Modelling*, 2, 06 2017.

[14] Amanda Minter and Renata Retkute. Approximate bayesian computation for infectious disease modelling. *Epidemics*, 29:100368, 2019.

[15] Mikael Sunnåker, Alberto Giovanni Busetto, Elina Numminen, Jukka Corander, Matthieu Foll, and Christophe Dessimoz. Approximate bayesian computation. *PLOS Computational Biology*, 9(1):1–10, 01 2013.

[16] Oxford COVID-19 Government Response Tracker. `https://www.bsg.ox.ac.uk/research/research-projects/coronavirus-government-response-tracker#data`, 2020. Accessed: 2020-11-30.

[17] Giancarlo De Luca, Kim Van Kerckhove, Pietro Coletti, Chiara Poletto, Nathalie Bossuyt, Niel Hens, and Vittoria Colizza. The impact of regular school closure on seasonal influenza epidemics: a data-driven spatial transmission model for Belgium. *BMC Infectious Diseases*, 18(1):29, 2018.

[18] Decreto del Presidente del Consiglio dei Ministri 3 novembre. `https://www.gazzettaufficiale.it/eli/id/2020/11/04/20A06109/sg`, 2020.

[19] ISTAT: Popolazione residente al 1 gennaio. `http://dati.istat.it/Index.aspx?DataSetCode=DCIS_POPRES1`, 2021. Accessed: 2021-07-13.

[20] Ana Pastore y Piontti, Nicola Perra, Luca Rossi, Nicole Samay, and Alessandro Vespignani. *Charting the Next Pandemic: Modeling Infectious Disease Spreading in the Data Science Age*. Springer, 2018.

[21] Matteo Chinazzi, Jessica T. Davis, Marco Ajelli, Corrado Gioannini, Maria Litvinova, Stefano Merler, Ana Pastore y Piontti, Kunpeng Mu, Luca Rossi, Kaiyuan Sun, Cécile Viboud, Xinyue Xiong, Hongjie Yu, M. Elizabeth Halloran, Ira M. Longini, and Alessandro Vespignani. The effect of travel restrictions on the spread of the 2019 novel coronavirus (covid-19) outbreak. *Science*, 368(6489):395–400, 2020.

[22] Report Vaccini Anti COVID-19. `https://www.governo.it/it/cscovid19/report-vaccini/`, 2021. Accessed: 2021-09-03.

[23] Stefan Elbe and Gemma Buckland-Merrett. Data, disease and diplomacy: Gisaid's innovative contribution to global health. *Global Challenges*, 1(1):33–46, 2017.

[24] Nicholas G Davies, Christopher I Jarvis, Kevin van Zandvoort, Samuel Clifford, Fiona Yueqian Sun, Sebastian Funk, Graham Medley, Yalda Jafari, Sophie R Meakin, Rachel Lowe, Matthew Quaife, Naomi R Waterlow, Rosalind M Eggo, Jiayao Lei, Mihaly Koltai, Fabienne Krauer, Damien C Tully, James D Munday, Alicia Showering, Anna M Foss, Kiesha Prem, Stefan Flasche, Adam J Kucharski, Sam Abbott, Billy J Quilty, Thibaut Jombart, Alicia Rosello, Gwenan M Knight, Mark Jit, Yang Liu, Jack Williams, Joel Hellewell, Kathleen O'Reilly, Yung-Wai Desmond Chan, Timothy W Russell, Simon R Procter, Akira Endo, Emily S Nightingale, Nikos I Bosse, C Julian Villabona-Arenas, Frank G Sandmann, Amy Gimma, Kaja Abbas, William Waites, Katherine E Atkins, Rosanna C Barnard, Petra Klepac, Hamish P Gibbs, Carl A B Pearson, Oliver Brady, W John Edmunds, Nicholas P Jewell, Karla Diaz-Ordaz, Ruth H Keogh, and CMMID COVID-19 Working Group. Increased mortality in community-tested cases of SARS-CoV-2 lineage B.1.1.7. *Nature*, 593(7858):270–274, 2021.

[25] Robert Challen, Ellen Brooks-Pollock, Jonathan M Read, Louise Dyson, Krasimira Tsaneva-Atanasova, and Leon Danon. Risk of mortality in patients infected with sars-cov-2 variant of concern 202012/1: matched cohort study. *BMJ*, 372, 2021.

[26] Aggiornamento indicazioni sulla Vaccinazione dei soggetti che hanno avuto un'infezione da SARS-CoV-2. `https://redas.services.siag.it/covidArticlesAttachment?attachId=1116555`, 2021.

[27] Hiroshi Nishiura, Tetsuro Kobayashi, Takeshi Miyama, Ayako Suzuki, Sung-mok Jung, Katsuma Hayashi, Ryo Kinoshita, Yichi Yang, Baoyin Yuan, Andrei R Akhmetzhanov, and Natalie M Linton. Estimation of the asymptomatic ratio of novel coronavirus infections (COVID-19). *International Journal of Infectious Diseases*, 94:154–155, may 2020.

[28] Enrico Lavezzo, Elisa Franchin, Constanze Ciavarella, Gina Cuomo-Dannenburg, Luisa Barzon, Claudia Del Vecchio, Lucia Rossi, Riccardo Manganelli, Arianna Loregian, Nicolò Navarin, Davide Abate, Manuela Sciro, Stefano Merigliano, Ettore De Canale, Maria Cristina Vanuzzo, Valeria Besutti, Francesca Saluzzo, Francesco Onelia, Monia Pacenti, Saverio G Parisi, Giovanni Carretta, Daniele Donato, Luciano Flor, Silvia Cocchio, Giulia Masi, Alessandro Sperduti, Lorenzo Cattarino, Renato Salvador, Michele Nicoletti, Federico Caldart, Gioele Castelli, Eleonora Nieddu, Beatrice Labella, Ludovico Fava, Matteo Drigo, Katy A M Gaythorpe, Alessandra R Brazzale, Stefano Toppo, Marta Trevisan, Vincenzo Baldo, Christl A Donnelly, Neil M Ferguson, Ilaria Dorigatti, Andrea Crisanti, Kylie E C Ainslie, Marc Baguelin, Samir Bhatt, Adhiratha Boonyasiri, Olivia Boyd, Lorenzo Cattarino, Constanze Ciavarella, Helen L Coupland, Zulma Cucunubá, Gina Cuomo-Dannenburg, Bimandra A Djafaara, Christl A Donnelly, Ilaria Dorigatti, Sabine L van Elsland, Rich

FitzJohn, Seth Flaxman, Katy A M Gaythorpe, Will D Green, Timothy Hallett, Arran Hamlet, David Haw, Natsuko Imai, Benjamin Jeffrey, Edward Knock, Daniel J Laydon, Thomas Mellan, Swapnil Mishra, Gemma Nedjati-Gilani, Pierre Nouvellet, Lucy C Okell, Kris V Parag, Steven Riley, Hayley A Thompson, H Juliette T Unwin, Robert Verity, Michaela A C Vollmer, Patrick G T Walker, Caroline E Walters, Haowei Wang, Yuanrong Wang, Oliver J Watson, Charles Whittaker, Lilith K Whittles, Xiaoyue Xi, Neil M Ferguson, and Imperial College COVID-19 Response Team. Suppression of a SARS-CoV-2 outbreak in the Italian municipality of Vo'. *Nature*, 584(7821):425–429, 2020.

[29] Nicola Perra. Non-pharmaceutical interventions during the covid-19 pandemic: A review. *Physics Reports*, 2021.

[30] Frederik Verelst, Lander Willem, and Philippe Beutels. Behavioural change models for infectious disease transmission: A systematic review (2010-2015). *Journal of The Royal Society Interface*, 13, 12 2016.

[31] Olga Krylova and David J. D. Earn. Effects of the infectious period distribution on predicted transitions in childhood disease dynamics. *Journal of The Royal Society Interface*, 10(84):20130098, 2013.
